# Levels and predictors of fear and health anxiety during the current outbreak of COVID-19 in immunocompromised and chronic disease patients in Saudi Arabia: A cross-sectional correlational study

**DOI:** 10.1371/journal.pone.0250554

**Published:** 2021-04-26

**Authors:** Jehan S. Al-Rahimi, Nada M. Nass, Shahira A. Hassoubah, Dhuha Y. Wazqar, Soha A. Alamoudi

**Affiliations:** 1 Department of Biological Sciences, Faculty of Sciences, Immunology Unit, King Fahd Medical Research Centre, King Abdulaziz University, Jeddah, Kingdom of Saudi Arabia; 2 Department of Biological Sciences, Faculty of Sciences, King Abdulaziz University, Jeddah, Kingdom of Saudi Arabia; 3 Department of Medical Surgical Nursing, Faculty of Nursing, King Abdulaziz University, Jeddah, Kingdom of Saudi Arabia; 4 Department of Biology, College of Science and Arts, King Abdulaziz University, Rabigh, Kingdom of Saudi Arabia; Imam Abdulrahman Bin Faisal University, SAUDI ARABIA

## Abstract

Coronavirus disease 2019 (COVID-19) infection has become a clinical threat to healthy people as well as immunocompromised patients and those with pre-existing chronic diseases around the world. This study, which used a cross-sectional correlational design, aimed to assess the levels of fear and health anxiety and to investigate their predictors during the current outbreak of COVID-19 in immunocompromised and chronic disease patients in Saudi Arabia. Sociodemographic and clinical data, fear of COVID-19, and health anxiety measurements were collected by online surveys from June 15 to July 15, 2020. Univariate and multiple linear regression analysis was used to identify predictors. A total of 1,030 patients in 13 provinces in Saudi Arabia completed the questionnaire. A significant number of patients with chronic diseases experienced considerable levels of fear and anxiety during the COVID-19 outbreak. It was found that 21.44% of participants met the criteria for anxiety cases, and 19.4% were considered borderline anxiety cases. In regression analysis, significant predictors of fear and health anxiety were female gender, lower education, middle-aged, divorced or widowed, receiving immunosuppressants, type of chronic disease (Crohn’s disease, hypertension, and cardiovascular diseases), and media use as a source of knowledge about COVID-19. Immunocompromised and chronic disease patients are vulnerable to fear and anxiety during epidemic infectious diseases such as COVID-19. Optimizing this population’s compliance with appropriate infection prevention and control strategies is crucial during the infectious outbreaks to ensure their safety, to decrease the risk of infection and serious complications, and reduce their fear and health anxiety. Effective positive psychological interventions and support strategies also need to be immediately implemented to increase psychological resilience and improve the mental health of these patients. Due to the COVID-19 outbreak, chronic disease patients in Saudi Arabia need special attention from health authorities, policymakers, and healthcare professionals to manage maladaptive forms of health anxiety and fear.

## 1. Introduction

Coronavirus disease 2019 (COVID-19), an infectious respiratory syndrome caused by the novel coronavirus SARS-CoV-2, has spread exponentially across 211 countries since January 2020, including the Middle East. The primary mode of COVID-19 transmission is by respiratory droplets and contact, putting anyone at high risk of exposure [1]. In June 15, 2020, more than 7 million COVID-19 cases and 431 541 deaths occurred around the world [2]. In Saudi Arabia (SA), the Ministry of Health (MOH) COVID-19 daily reporting as of June 14, 2020, revealed a total of 131,774 confirmed cases among Saudis [3]. As of July 16, 2020, it reported 243,238 confirmed cases with 2,370 deaths, the highest among the Arab states of the Persian Gulf [3]. The WHO formally declared the COVID-19 outbreak a public emergency on January 30, 2020, and a global pandemic on March 11, 2020 [4, 5]. Immunocompromised and chronic disease patients are under tremendous psychological and physical stress in the face of this large-scale infectious public health crisis.

Immunocompromised patients and those with pre-existing chronic diseases such as cancer, diabetes, hypertension, cardiovascular and renal diseases, and respiratory disorders tend to be at greater risk of developing serious complications and are at high risk of death due to COVID-19 infection [6–8]. Liang et al. [8] indicated that cancer patients are more vulnerable to infection than those without cancer due to their state of systemic immunosuppression, caused by cancer itself and treatment modalities such as radiotherapy or chemotherapy. Guo et al. [7] stated that COVID-19 infection can alter the course of the underlying disease in patients with cardiovascular problems and can increase mortality because the overall stress caused by this infection can affect the cardiac muscle. According to preliminary estimates in the United States, around one-third of infected patients with COVID-19 (2,692, 38%), had at least one chronic disease or risk factor; the most common were cardiovascular diseases (647, 9%), chronic lung diseases (656, 9.2%), and diabetes (784, 11%) [6]. SA is a leading country with a high prevalence of chronic diseases (ischemic heart disease, hypertension, chronic kidney disease, diabetes, asthma, gastric/peptic ulcer, and cancers) due to rapid economic development, urbanization, smoking, and changes in lifestyle patterns [9–11]. It accounted for 75% of cancer cases in a 12-year cancer prevalence report of Gulf Cooperation Council states and ranked seventh in the world for its diabetes rate [12, 13]. However, data on the prevalence of chronic conditions among Saudi patients with COVID-19 have not yet been published. The increasing number of confirmed cases, a lack of knowledge of COVID-19, the rapid transmission rate, lockdown conditions, difficulties in routine medical treatments, shortages of human resources in hospitals, insufficient psychological preparation, separation from loved ones, loss of freedom, and uncertainty over illness status may cause anxiety, emotional disturbance, confusion, depression, and fear among immunocompromised and chronic disease patients during the outbreak [14–16]. Anxiety and panic, caused by stress and loneliness, may also be common among these patients leading to negative effects [15].

Since the outbreak of COVID-19, few studies outside of SA have examined the psychological impact on immunocompromised and chronic diseases patients and found that they experienced psychological disorders such as depression, stress, and anxiety [15, 17]. However, the sample sizes of early surveys have been comparatively small (<400 participants) because of the limited time-frame of the investigations [15, 17]. Moreover, uncertainty and fear are common in any disease outbreak, and therefore, appropriate infection prevention and control strategies and effective mental health and medical interventions are essential for this highly vulnerable population. Therefore, the current study aimed to assess the levels of fear and health anxiety during the current outbreak of COVID-19 in SA in immunocompromised and chronic disease patients. It further aimed to investigate possible predictors (sociodemographic and clinical factors, sources of knowledge about COVID-19, and media exposure) of increased fear and health anxiety regarding COVID-19.

## 2. Materials and methods

### 2.1. Study design and participants

This cross-sectional correlational study was conducted from June 15 to July 15, 2020, in a period when the Saudi MOH reported a high number of COVID-19 cases, including critical cases, and precautionary strict measures were imposed by several Middle Eastern countries, and fear and worry towards this emerging infection was increasing among the public. Various measures were taken by the Saudi government to reduce the spread of the virus during the pandemic (at the period of data collection) such as lockdowns, social distancing, restrictions on public gatherings and internal movements, and self-isolation. Anonymous Online Arabic questionnaires, using a Google Forms Survey to ensure easy access and wide reach, were sent to people throughout the country via popular professional networks and social media platforms (email, Facebook, Twitter, LinkedIn, and WhatsApp). Immunocompromised and chronic disease patients were contacted through sending private recruitment messages, asking for referrals, posting/tweeting in chronic disease discussion groups, posting Facebook-targeted ad, tagging users, chronic patient support groups, and Saudi health organizations/society who have a large following, as well as asking some social media influencers to post the study advertisement to their followers. Convenience and snowball sampling methods were used to recruit participants, and a total of 1,030 immunocompromised and chronic disease patients from 13 provinces in SA were surveyed. The questionnaire included general sociodemographic characteristics (age, gender, nationality, marital status, number of children, education, place of residence, employment status, and monthly income), clinical data (smoking history [non-smoker or active smoker; currently smoked at least one cigarette a day], type of chronic diseases, whether taking immunosuppressants or received antibiotics recently), fear, and hospital anxiety. Furthermore, data were collected on sources of knowledge about COVID-19 (MOH website, social media, radio, television, newspaper, and friends) and hours per day of media exposure receiving information about COVID-19 in the last week. Participation was voluntary and prior written informed consent online was provided by all study participants (information regarding participation, confidentiality, and informed consent were written clearly in the online submission information). Answers to all questions were mandatory, and participants were unable to submit their answers if any question was left unanswered, to avoid any missing data. Also, while creating the Google Form Survey, we turned on the option that says *“*Allow only one response per user*”* to avoid the questionnaire being answered by the same participant more than once. During the data cleaning process, any replicate submissions that we might expect from a single person were deleted before starting the actual analysis. Also, in the Google Form Survey we created screening questions to screen out people who did not meet the inclusion criteria. Based on this, the eligible participants could only move on to next page to complete the study survey. The Research Ethics Committee of the Faculty of Nursing at King Abdulaziz University Hospital reviewed and approved this study (No. 2020REC03).

According to Green [18], in regression studies, the minimum sample size needed with eight additional observations per term should be 50, where *n* ≥ 50 + 8*m*, with *m* being the number of predictors. Therefore, a sample size of 138 participants would be required in this study for multiple regression analysis with 11 independent variables. However, for a non-experimental clinical study, a larger sample size of 300 or more is necessary [19]. Bullen indicates that most statisticians agree that a minimum sample size of 100 is needed to obtain some kind of meaningful result and that if the population more than 5000, the maximum sample size is 1000, which will usually yield a reasonably accurate result [20]. To increase the statistical power for strengthening the findings’ robustness and detecting smaller effects, we allowed a larger sample size in this study. Eligibility criteria were (a) being aged over 18 years, (b) speaking Arabic as a first language, (c) living in SA, whether Saudi or non-Saudi, (d) being diagnosed with a chronic disease (cancer, hypertension, diabetes, asthma, or cardiovascular or autoimmune disease) or currently receiving immunosuppressants (steroids), and (e) not being infected with COVID-19. Otherwise eligible people were excluded if they were on psychotropic medications or had psychiatric or neurological disorders.

### 2.2 Measures

The fear of participants was measured by the Fear of COVID-19 Scale (FCV-19S) Arabic version (see S1 Appendix). The FCV-19S [21] is a 7-item self-report instrument developed to measure individuals’ fear levels of COVID-19, rated on a 5-point Likert scale (ranging from 1—strongly disagree to 5—strongly agree). For each question, the minimum possible score is 1 and the maximum is 5. To score the FCV-19S, all items are summed to obtain the fear score (ranging from 7 to 35). A higher score reflects greater levels of fear of COVID-19. This scale takes under 5 minutes to complete. The FCV-19S has shown reliability and validity in measuring the fear levels of COVID-19 in the general public, with an internal consistency estimate of 0.82 and test–retest reliability (Intra-class Correlation Coefficients [ICC]) of 0.72 [21]. The scale is currently available in four languages: Arabic, Turkish, Italian, and Bangla [22–25]. The Cronbach’s alpha value for the FCV-19S Arabic version in the current sample was 0.87.

The Hospital Anxiety and Depression Scale (HADS) is a 14-item self-report instrument developed to assess anxiety and depression levels, comprising a 7-item measure of depression (HADS-D) and a 7-item measure of anxiety (HADS-A) [26]. Each item has four possible answers (0–3), and scores range from 0 to 21 for each subscale. A score of 11–21 is considered a case (anxiety or depression), 8–10 a borderline case, and 0–7 normal. The HADS has been validated by item response theory and both confirmatory and exploratory factor analyses. The Cronbach’s alpha ranges from 0.68 to 0.93 for HADS-A and from 0.67 to 0.90 for HADS-D [26]. The HADS is available in many languages including Arabic (see S1 Appendix) [27]. The Cronbach’s alpha for the overall HADS-A Arabic version in the current sample was 0.86.

### 2.3 Statistical analysis

Statistical analyses were performed on IBM SPSS version 24 (IBM, Inc., Armonk, NY, USA). Descriptive results were expressed as the frequency with percentage (%), mean, and standard deviation (SD). To assess the normal distribution of data, the Kolmogorov–Smirnov test was used. Predictors of COVID-19 fear as assessed by the FCV-19S and health anxiety as assessed by the HADS-A were investigated using one-way ANOVA for the categorical predictors and simple Pearson’s correlation coefficients for the continuous predictors, with all predictors included. Multiple linear regression analysis was performed, comprising predictors that were significant in the univariate analyses, to examine the relative contribution of each predictor in explaining variance in the increased fear and health anxiety regarding COVID-19. The FCV-19S and HADS-A were considered dependent variables, whereas age, gender, marital status, education, type of chronic disease, taking immunosuppressants, received antibiotics recently, and sources of knowledge about COVID-19 were treated as independent variables. Two regression analyses were conducted: the first with fear (FCV-19S) as the dependent variable and the second with health anxiety (HADS-A) as the dependent variable. For all analyses, the level of significance was set at *P* < 0.05.

## 3. Results

### 3.1 Participant sociodemographic and clinical characteristics

A total of 1,030 individuals submitted the online survey; 76.1% (n = 784) of the participants were women and 23.9% (n = 246) were men. The means of the participant age, number of children, and monthly income were 36.4±0.39 (range: 20–80) years, 2.21±0.109 (range: 0–7), and 10,527±2445.71 Saudi riyals, respectively. Most participants (88.4%) were Saudi, and 96.3% lived in urban areas. Approximately 61% of participants were married, and 41.1% had a bachelor’s degree. Forty-eight percent of the participants were employed, in either the government or private sectors. Only 25.1% were active smokers, and 29.6% were receiving one or a combination of immunosuppressant medications. Further, 14.6% of the participants had received antibiotics recently (in the last month). Participants with a diagnosis of hypertension or other cardiovascular diseases accounted for around one-third of the sample (29%), and the rest were diagnosed with cancer (27.4%), rheumatoid arthritis (19.5%), diabetes (12.8%), and other chronic diseases (11.4%), such as asthma, Crohn’s disease, and thyroid problems. The commonest source of knowledge about COVID-19 was the MOH website (55.5%), and the median level of media exposure to COVID-19 was 3 hours/day (See Table 1).

**Table 1:** Sociodemographic and clinical characteristics of the participants.

Characteristic	n (%)
Age (Year), range	
20–30	131 (12.71)
31–40	253 (24. 54)
41–50	328 (31.81)
>50	319 (30.94)
Gender	
Male	246 (23.9)
Female	784 (76.1)
Nationality	
Saudi	911 (88.4)
Non-Saudi	119 (11.6)
Marital status	
Single	337 (32.7)
Married	625 (60.7)
Divorced/separated	47 (4.6)
Widow	21 (2.0)
Number of children, range	
Area of residence	
Urban	992 (96.3)
Rural	38 (3.7)
Level of education	
Elementary [First stage of Saudi formal education; grades one to six]	65 (6.3)
Intermediate [Second stage of Saudi formal education; grades seven to nine]	156 (15.1)
Secondary [Third stage of Saudi formal education; grades 10 to 12]	174 (16.9)
Diploma	118 (11.5)
Bachelor degree	423 (41.1)
Master/PhD degree	94 (9.1)
Employment status	
Student	209 (20.3)
Employed	495 (48.1)
Unemployed	244 (23.7)
Retired	82 (8.0)
Monthly income (Saudi Riyals), range	
< 5000	503 (48.8)
5000–10000	247 (24.9)
11000–15000	137 (13.3)
16000–20000	77 (7.5)
>20000	66 (6.4)
Smoking history	
Yes	259 (25.1)
No	771 (74.9)
Type of Chronic disease	
Asthma	89 (8.6)
Cancer	282 (27.4)
Cardiovascular diseases	137 (13.3)
Diabetes	132 (12.8)
Crohn’s disease	17 (1.7)
Hypertension	161 (15.6)
Rheumatoid arthritis	201 (19.5)
Others*	11 (1.1)
Taking immunosuppressants	
Yes	305 (29.6)
No	725 (70.4)
Taking antibiotics recently (in the last month)	
Yes	150 (14.6)
No	880 (85.4)
Source of knowledge about COVID-19	
Ministry of Health Website (MOH)	572 (55.5)
Social media	325 (31.6)
TV/radio	119 (11.6)
Newspaper	4 (0.4)
Friends	10 (1.0)
Media exposure to COVID-19 (hours/day)	
1	201 (19.5)
2	233 (22.6)
3	304 (29.5)
4	146 (14.2)
≥ 5	145 (14.1)
Total	1030 (100)

*Others, systemic lupus erythematosus, Kidney failure, thyroid problems, liver cirrhosis, colon diseases.

### 3.2 Fear of COVID-19 and heath anxiety descriptive analysis

The mean FCV-19S score was 17.40±5.751, with scores ranging from 7 to 35, which indicates that participants in this study experienced a considerable level of fear of COVID-19. A total of 41.9% of participants mentioned that they felt uncomfortable thinking about COVID-19. More than one-third (35.6%) of participants reported that they felt nervous or anxious when they watched stories and news about COVID-19 on social networks. Additionally, 28.3% agreed that they were most afraid of COVID-19 (See Table 2).

**Table 2:** Descriptive statistics of patients’ fear of COVID-19 and hospital anxiety and depression scales.

**Fear of COVID-19 Scale Item**	**Strongly disagree *n* (%)**	**Disagree *n* (%)**		**Neutral *n* (%)**		**Agree *n* (%)**		**Strongly agree *n* (%)**
I am most afraid of Corona	121 (11.7)	218 (21.2)		305 (29.6)		292 (28.3)		94 (9.1)
It makes me uncomfortable to think about Corona	76 (7.4)	176 (17.1)		210 (30.4)		432 (41.9)		136 (13.2)
My hands become clammy when I think about Corona	555 (53.9)	349 (33.9)		82 (8.0)		35 (3.4)		9 (0.9)
I am afraid of losing my life because of Corona	299 (29.0)	278 (27.0)		194 (18.8)		176 (17.1)		83 (8.1)
When I watch news and stories about Corona on social media, I become nervous or anxious	120 (11.7)	209 (20.3)		215 (20.9)		367 (35.6)		119 (11.6)
I cannot sleep because I’m worrying about getting Corona	535 (51.9)	318 (30.9)		116 (11.3)		49 (4.8)		12 (1.2)
My heart races or palpitates when I think about getting Corona	441 (42.8)	292 (28.3)		153 (14.9)		119 (11.6)		25 (2.4)
Total (7 questions)	Mean 17.40± 5.751							
**Hospital Anxiety and Depression Item**	**Most of the time *n* (%)**		**A lot of the time *n* (%)**		**Occasionally *n* (%)**		**Not at all *n* (%)**	
I feel tense or ’wound up’	120 (11.7)		207 (20.1)		498 (48.3)		205 (19.9)	
I get a sort of frightened feeling as if something awful is about to happen	117 (11.4)		266 (25.8)		426 (41.4)		221 (21.5)	
Worrying thoughts go through my mind	70 (6.8)		203 (19.7)		431 (41.8)		326 (31.7)	
I cannot sit at ease and feel relaxed	41 (4.0)		338 (32.8)		254 (24.7)		397 (38.5)	
I get a sort of frightened feeling like ’butterflies’ in the stomach	69 (6.7)		108 (10.5)		589 (57.2)		264 (25.6)	
I feel restless as I have to be on the move	46 (4.5)		87 (8.4)		372 (36.1)		525 (51.0)	
I get sudden feelings of panic	51 (5.0)		111 (10.8)		338 (32.8)		530 (51.5)	
Total (7 questions)	Mean 6.89±4.537							

Fear of COVID-19 Scale; Scoring: 1 = Strongly disagree; 2 = Disagree; 3 = Neutral; 4 = Agree; 5 = Strongly agree. Range: 7 to 35, higher scores reflect greater levels of fear of COVID-19.

Hospital Anxiety and Depression Scale; Scoring: 0 = Not at all; 1 = Occasionally; 2 = A lot of the time; 3 = Most of the time. Range: 0 to 21, 11–21 is considered a case of anxiety.

The mean HADS-A score was 6.89±4.537, with scores ranging from 0 to 21, and 21.44% of participants were identified as anxiety cases. Notably, 19.4% of participants were considered borderline anxiety cases, and 59.2% had a normal level of health anxiety. Around 38.5% reported they could not sit at ease and did not feel relaxed, 25% experienced frightened feelings, and 19.7% had worrying thoughts in their minds (See Table 2).

### 3.3 Factors associated with fear and health anxiety

The results of the univariate analyses investigating the possible predictors of the FCV-19S and HADS-A shows that female gender, marital status, level of education, type of chronic disease, taking immunosuppressants, receiving antibiotics recently, and sources of knowledge about COVID-19 were significantly associated with increased fear of COVID-19. Fear scores were significantly higher among women (mean 17.99±5.67), divorced (mean 18.81±5.31) or widowed (mean 18.33±6.77) participants, those with lower education (mean 21±7.07), participants receiving immunosuppressants (mean 19.56±5.28), and those with hypertension (mean 31.67±5.77) and cardiovascular diseases (mean 20.76±5.25). Health anxiety scores were significantly higher among those between the ages of 31 and 40 years (mean 7.59±4.78), women (mean 7.19±4.45), participants receiving immunosuppressants (mean 9.62±4.38), who received antibiotics recently (mean 8.08±4.91), and those with Crohn’s disease (mean 10.11±4.40). The other predictors (nationality, area of residence, employment status, number of children, monthly income, smoking, and media exposure to COVID-19) were not predictive of increased COVID-19 fear and health anxiety. The correlation between health anxiety and fear of COVID-19 was also significant, strong, and positive (*r* = 0.641, *P* < 0.001).

Table 3 shows the predictors of COVID-19 fear and health anxiety based on multiple linear regression analyses. In regression model 1, the significant predictors of fear of COVID-19 were female gender (β = .109, *P* < 0.001), marital status (β = .057, *P* = 0.016), type of chronic disease (β = .038, *P* = 0.029), taking immunosuppressants (β = -.001, *P* = 0.036), sources of knowledge about COVID-19 (β = .028, *P* = 0.008), and health anxiety (β = .625, *P* < 0.001). The R2 (AR2) of the regression model 1 was .427 (.423), F = 126.93, and *P* < 0.001; this model explained 43% of the variance in the FCV-19S. According to regression model 2, age (β = -.112, *P* = 0.001), type of chronic disease (β = .089, *P* < 0.001), taking immunosuppressants (β = -.066, *P* = 0.007), recently receiving antibiotics (β = -.067, *P* = 0.005), and fear of COVID-19 (β = .622, *P* < 0.001) were found to predict health anxiety. The R2 (AR2) of the regression model 2 was .433 (.429), F = 111.426, *P* < 0.001.

**Table 3:** Predictors of fear and health anxiety regarding COVID-19 in multiple linear regression analysis.

Variable	Fear of COVID-19 (FCV-19S) Model 1					Health Anxiety (HADS-A) Model 2				
	*B*	*SE*	β	*t*	*P*	*B*	*SE*	β	*t*	*P*
Age						-0.041	0.01	-0.112	-3.476	0.001
Gender	1.469	0.323	0.109	4.433	<0.001	-0.163	0.263	-0.015	-0.662	0.534
Marital status	0.522	0.217	0.057	1.802	0.016					
Taking immunosup-pressants	-0.026	0.640	-0.001	-0.298	0.036	-1.383	0.508	-0.066	-2.724	0.007
Received antibiotics	0.191	0.390	0.012	0.432	0.625	-0.866	0.306	-0.067	-2.827	0.005
Type of chronic disease	0.072	0.048	0.038	1.517	0.029	0.133	0.037	0.089	3.587	<0.001
Source of knowledge	-0.205	0.175	-0.028	-1.172	0.008					
Fear						0.491	0.019	0.622	25.614	<0.001
Health anxiety	0.792	0.031	0.625	25.614	<0.001					

B, unstandardized regression weight, SE, Standard error, β, Standardized regression coefficients, AR2, adjusted R2.

Model 1: R2 (AR2) = .427 (.423), F (*P*) = 126.93 (< 0.001).

Model 2: R2 (AR2) = .433 (.429), F (*P*) = 111.426 (< 0.001).

## 4. Discussion

In this cross-sectional correlational study of 1,030 immunocompromised and chronic disease patients, the majority of participants experienced a certain level of fear of acquiring the infection, 21.44% were identified as anxiety cases, and 19.4% were considered borderline anxiety cases during the outbreak of COVID-19 in SA. Also, it is clear that the participants show considerable levels of fear and health anxiety as a group, and the levels reported in this study are above the cut-off points identified for each of these variables in the previous studies (using the same scales; FCV-19S and HADS-A) in healthy adult populations [28, 29]. In general, women and older patients with chronic diseases who were receiving immunosuppressants were more likely to have high levels of fear and health anxiety. The type of chronic disease and sources of COVID-19 knowledge were also associated with increased levels of fear and health anxiety. The type of chronic disease seemed to be a significantly strong predictor of the levels of fear and health anxiety reported by participants. Those who were divorced or unmarried were more likely to have a high level of fear, but less likely to have health anxiety. Our results are consistent with previous studies that have found more signs of fear and anxiety in individuals with chronic diseases than those without chronic diseases during a pandemic [17, 19, 30]. Health anxiety was strongly associated with increased fear of the current COVID-19 pandemic and one of the fear predictors among this population. This suggests that health anxiety is a unique factor explaining the fear of COVID-19 more than other general scales of worry and anxiety. This result replicates findings from an online study conducted in March 2020 investigating predictors of fear of COVID-19 [31]. However, we have not encountered studies in the literature that examined fear and health anxiety related to COVID-19 in immunocompromised and chronic disease patients in SA to compare and contrast with the results of this study.

This study also demonstrated that female patients with chronic diseases are more likely to have high levels of fear and health anxiety, which is consistent with two previous studies conducted in Iran and Turkey during the COVID-19 outbreak [32, 33], as well as findings of earlier research on women’s mental health [34]. Gender differences exist in health and mental status [35]. In contrast, Gorrochategi et al. [15] found that men with chronic diseases in Spain experienced more anxiety than women with chronic diseases. Possible explanations for this difference may be the selection criteria (people aged 60−65 years) and the small sample size (250). The present study found that divorced or widowed as well as middle-aged adult patients with chronic diseases experienced high levels of fear and heath anxiety, consistent with previous research [17]. Middle-aged people with chronic diseases may have higher chronic disease and family burdens and may be more worried about their health and family members, which in turn impact their psychological wellbeing. Divorced or widowed people with chronic diseases may receive inadequate care or communication and a low level of family support. Lam and Perales [36] indicated that the magnitude of the interaction effects was not negligible between marital status, mental health, and chronic illnesses. However, our finding further highlights the importance of social support services and facilities for people with chronic diseases to sustain good mental health. Healthcare providers should also design and implement effective mental health interventions to protect the psychological health of those with chronic health problems during infectious disease outbreaks.

The levels of fear and health anxiety were high in participants who had autoimmune diseases or were receiving immunosuppressants or received antibiotics recently. This may be because they are expected to be at higher risk of infection and have serious symptoms and complications if infected or have antibiotics resistance if they develop a serious secondary bacterial infection. Previous studies conducted among immunocompromised patients and those with chronic conditions during the COVID-19 outbreak have also shown that they were more likely to develop distress, panic attacks, worry, and anxiety and to use behavioral detachment than the general population [37, 38]. These fears and sources of anxiety should be addressed through support targeting crisis-specific needs of immunocompromised people and those with chronic diseases, as well as regular updates of information on COVID‐19 and chronic diseases. The proper information to increase their awareness about infection prevention and control methods are also required.

The current study also showed a significant relationship between sources of COVID-19 knowledge and fear of COVID-19, in line with two studies examining sources of and exposure to media information about COVID-19 [31, 39]. We found that the official website of the Saudi MOH, a reliable source of information, was used more than social media and other sources for COVID-19 information. Based on this finding, reporters, policymakers, and healthcare professionals have opportunities to control excessive fear among patients with chronic diseases as well as the general population by using their official platform to promote mental health (e.g. provide awareness massages, preventive guidelines, and measures targeting various groups) and provide mental health services (e.g. online consultation support) during the period of the COVID-19 outbreak. Such information and mental health services may play a role in decreasing the level of fear of COVID-19 in those with chronic diseases during infectious disease outbreaks.

One of the main limitations of the study is the possibility of selection bias as this study was conducted with an online survey. Immunocompromised and chronic disease patients who did not have internet access and were unable to use social media, email, or cell phones were not involved in the study. Another limitation of the current study includes its cross-sectional design (limited in its ability to conclude associations and long-term effects) and as the epidemic changes, the mental health of this population may also change. More studies are required to track the dynamic changes of immunocompromised and chronic disease patients’ psychological state during the pandemic. We included in this study only those who have chronic diseases or currently receiving immunosuppressants in SA, it would be desirable in future research to compare the healthy population’s psychological responses in this context. Also, we included only a limited set of possible predictors (sociodemographic and clinical variables, sources of knowledge about COVID, and media exposure). Further research on fear and health anxiety and their associated factors among immunocompromised and chronic disease patients in the COVID-19 pandemic examining associated-variables that were not included in our study is recommended: perceptions of infection risk and beliefs regarding COVID-19, intolerance of uncertainty, precautionary behaviors, compliance with preventive measures, and access to health care services and disruption in treatment. The present study included more women than men which means that our interpretation of gender as a variable is limited and potentially resulting in an underestimation of fear and health anxiety levels of male chronic disease patients. Although we tried to distribute the questionnaire fairly across our personal and professional networks and social media, research indicates that gender influences web-based survey participation [40]. Finally, this type of self-report questionnaire introduces the possibility of social desirability bias among participants.

## 5. Conclusions

This study assessed the levels of fear and health anxiety and investigated their possible predictors during the current outbreak of COVID-19 in SA in immunocompromised and chronic disease patients. We found that a considerable number of immunocompromised and chronic disease patients experienced fear and heath anxiety in the current COVID-19 outbreak. Female, divorced or widowed, and middle-aged adult patients with chronic diseases were more vulnerable to have high levels of fear and health anxiety, which may lead to poor mental and physical health. The official website of the Saudi MOH was the most trusted information source of COVID-19 knowledge, while social media was one of the primary knowledge sources. Our findings can help in the development of interventions to mitigate the negative psychological effects of the COVID-19 pandemic on chronic disease patients in SA in the future.

## 6. Recommendations

Optimizing chronic disease patients’ compliance with appropriate infection prevention and control strategies is crucial during infectious outbreaks to ensure their safety, to decrease the risk of infection and serious complications, and reduce their fear and health anxiety. Effective positive psychological interventions and support strategies also need to be immediately implemented to increase psychological resilience and improve the mental health of these patients with psychological issues. Further, a multidisciplinary mental health approach including evaluation, education, and coaching will be required to decrease levels of fear and health anxiety in this population during infectious disease outbreaks. For most immunocompromised and chronic disease patients, behavioral and emotional reactions are elements of an adaptation to a higher stress level, and psychotherapy approaches established on the stress-adaptation model may be useful [41]. Health authorities, policymakers, and healthcare professionals, including nurses, in SA and other countries should give special attention to patients with chronic diseases, to manage maladaptive forms of anxiety and fear due to the COVID-19 outbreak.

## Supporting information

S1 Appendix A copy of the English and Arabic versions of the questionnaire used in study with references. (DOCX)

## References

[1] Li Q, Guan X, Wu P, Wang X, Zhou L, Tong Y et al. Early transmission dynamics in Wuhan, China, of novel Coronavirus–infected pneumonia. N Engl J Med. 2020; 382(19): 1199–1207. 10.1056/NEJMoa2001316 31995857 PMC7121484

[2] World Health Organization (WHO). Coronavirus disease 2019 (COVID-19) Situation Report-147. Available from: https://www. https://www.who.int/docs/default-source/coronaviruse/situation-reports/20200615-covid-19-sitrep-147.pdf?sfvrsn=2497a605_4. Accessed 19 January 2021. 2020.

[3] Abueish T. Saudi Arabia reports 4,233 new cases, highest daily rise so far. Al Arabiya. Available from: https://english.alarabiya.net/en/coronavirus/2020/06/14/Coronavirus-Saudi-Arabiareports-4-233-new-cases-40-COVID-19-deaths.html. Accessed 22 June 2020.2020.

[4] Cucinotta D, Vanelli M. WHO declares COVID-19 a pandemic. Acta Biomedica. 2020; 91(1): 157–160. 10.23750/abm.v91i1.9397 32191675 PMC7569573

[5] World Health Organization (WHO). Statement on the second meeting of the International Health Regulations (2005) Emergency Committee regarding the outbreak of novel coronavirus (2019-nCoV). Available from: https://www.who.int/news/item/30-01-2020-statement-on-the-second-meeting-of-the-international-health-regulations-(2005)-emergency-committee-regarding-the-outbreak-of-novel-coronavirus-(2019-ncov). Accessed 2 June 2020. 2020.

[6] CDC COVID-19 Response Team. Preliminary estimates of the prevalence of selected underlying health conditions among patients with coronavirus disease 2019-United States, February 12–March 28, 2020. Morb Mortal Wkly Rep. 2020; 69(13): 382–386. 10.15585/mmwr.mm6913e2. PMC7119513 32240123

[7] Guo T, Fan Y, Chen M, Wu X, Zhang L, He T et al. Cardiovascular implications of fatal outcomes of patients with coronavirus disease 2019 (COVID-19). JAMA Cardiol. 2020; 5(7): 811–818. 10.1001/jamacardio.2020.1017 32219356 PMC7101506

[8] Liang W, Guan W, Chen R, Wang W, Li J, Xu K et al. Cancer patients in SARS-CoV-2 infection: a nationwide analysis in China. The Lancet Oncol. 2020; 21: 335–337. 10.1016/S1470-2045(20)30096-6 32066541 PMC7159000

[9] Memish ZA, Jaber S, Mokdad AH, AlMazroa MA, Murray CJ, Al Rabeeah AA. Burden of disease, injuries, and risk factors in the Kingdom of Saudi Arabia, 1990–2010. Prev Chronic Dis. 2014; 11 (E169): 140176. 10.5888/pcd11.140176. PMC4184091 25275806

[10] Saquib N, Saquib J, Alhadlag A, Albakour MA, Aljumah B, Sughayyir M et al. Chronic disease prevalence among elderly Saudi men. Int. J. Health Sci. 2017; 11(5), 11–16. https://ijhs.org.sa/index.php/journal/article/view/2047. PMC5669504 29114188

[11] The Institute for Health Metrics and Evaluation (IHME). Saudi Arabia. Available from: http://www.healthdata.org/saudi-arabia. Accessed 19 January 2021. 2020.

[12] Al Dawish MA, Robert AA, Braham R, Al Hayek AA, Al Saeed A, Ahmed RA et al. Diabetes mellitus in Saudi Arabia: a review of the recent literature. Curr Diabetes Rev. 2016; 12(4): 359–368. 10.2174/1573399811666150724095130 26206092

[13] Jazieh AR, Da’ar OB, Alkaiyat M, Zaatreh YA, Saad AA, Bustami R et al. Cancer incidence trends from 1999 to 2015 and contributions of various cancer types to the overall burden: projections to 2030 and extrapolation of economic burden in Saudi Arabia. Cancer Manag Res. 2019; 2019(11): 9665–9674. 10.2147/CMAR.S222667 32009819 PMC6861167

[14] Chudasama YV, Gillies CL, Zaccardi F, Coles B, Davies MJ, Seidu S et al. Impact of COVID-19 on routine care for chronic diseases: a global survey of views from healthcare professionals. Diabetes Metab Syndr. 2020; 14(5): 965–967. 10.1016/j.dsx.2020.06.042 32604016 PMC7308780

[15] Gorrochategi MP, Munitis ME, Santamaria MD, Etxebarria NO. Stress, anxiety, and depression in people aged over 60 in the COVID19 outbreak in a sample collected in northern Spain. Am J Geriatr Psychiatry. 2020. Available from: 10.1016/j.jagp.2020.05.022. PMC7261426 32576424

[16] Pellino G, Spinelli A. How COVID-19 outbreak is impacting colorectal cancer patients in Italy: a long shadow beyond infection. Dis Colon Rectum. 2020; 63(6): 720–722. 10.1097/DCR.0000000000001685 32384401

[17] Özdin S, Bayrak Özdin Ş. Levels and predictors of anxiety, depression and health anxiety during COVID-19 pandemic in Turkish society: the importance of gender. The International J Soc Psychiatry. 2020; 66(5): 504–511. 10.1177/0020764020927051 32380879 PMC7405629

[18] Green SB. How many subjects does it take to do a regression analysis. Multivariate Behav Res. 1991; 26(3): 499–510. 10.1207/s15327906mbr2603_7 26776715

[19] Bujang MA, Sa’at N, Sidik TMA. Determination of minimum sample size requirement for multiple linear regression and analysis of covariance based on experimental and non-experimental studies. Epidemiol Biostat Public Health. 2017; 14(3): e12117-1–e12117-9. 10.2427/12117.

[20] Bullen PB. How to choose a sample size (for the statistically challenged). Available from: http://www.tools4dev.org/resources/how-to-choose-a-sample-size/. Accessed 10 March 2021. 2014.

[21] Ahorsu DK, Lin CY, Imani V, Saffari M, Griffiths MD, Pakpour AH. The fear of COVID-19 scale: development and initial validation. Int J Ment Health Addict. 2020. Available from: 10.1007/s11469-020-00270-8 32226353 PMC7100496

[22] Alyami M, Henning M, Krägeloh CU, Alyami H. Psychometric evaluation of the Arabic version of the fear of COVID-19 scale. Int J Ment Health Addict. 2020. Available from: 10.1007/s11469-020-00316-x 32427217 PMC7229877

[23] Satici B, Gocet-Tekin E, Deniz ME, Satici SA. Adaptation of the fear of COVID-19 scale: its association with psychological distress and life satisfaction in Turkey. Int J Ment Health Addict. 2020. Available from: 10.1007/s11469-020-00294-0 32395095 PMC7207987

[24] Soraci P, Ferrari A, Abbiati FA, Del Fante E, De Pace R, Urso A et al. Validation and psychometric evaluation of the Italian version of the Fear of COVID-19 Scale. Int J Ment Health Addict. 2020. Available from: 10.1007/s11469-020-00277-1 32372892 PMC7198091

[25] Sakib N, Bhuiyan AKMI, Hossain S, Al Mamun F, Hosen I, Abdullah AH et al. Psychometric validation of the Bangla Fear of COVID-19 Scale: confirmatory factor analysis and Rasch analysis. Int J Ment Health Addict. 2020; 1–12. Available from: 10.1007/s11469-020-00289-x 32395096 PMC7213549

[26] Zigmond AS, Snaith RP. The Hospital Anxiety and Depression Scale. Acta Psychiatr Scand. 1983; 67(6): 361–370. 10.1111/j.1600-0447.1983.tb09716.x 6880820

[27] Terkawi AS, Tsang S, AlKahtani GJ, AlKahtani GJ, Al-Mousa SH, Al Musaed S et al. Development and validation of Arabic version of the Hospital Anxiety and Depression Scale. Saudi J Anaesth. 2017; 11(Suppl 1): S11–S18. 10.4103/sja.SJA_43_17 28616000 PMC5463562

[28] Rodríguez-Hidalgo AJ, Pantaleón Y, Dios I, Falla D. Fear of COVID-19, stress, and anxiety in university undergraduate students: a predictive model for depression. Front Psychol. 2020; 11: 591797. 10.3389/fpsyg.2020.591797 33224080 PMC7674167

[29] Alyami M, Albuquerque JV, Krägeloh CU, Alyami H, Henning MA. Effects of fear of COVID-19 on mental well-being and quality of life among Saudi adults: a path analysis. Saudi J Med Med Sci. 2021; 9(1): 24–30. 10.4103/sjmms.sjmms_630_20 33519340 PMC7839565

[30] Karacin C, Bilgetekin I, Basal FB, Oksuzoglu OB. How does COVID-19 fear and anxiety affect chemotherapy adherence in patients with cancer. Future Oncol. 2020; 16(29): 2283–2293. 10.2217/fon-2020-0592 32677462 PMC7367513

[31] Mertens G, Gerritsen L, Duijndam S, Salemink E, Engelhard IM. Fear of the coronavirus (COVID-19): predictors in an online study conducted in March 2020. J Anxiety Disord. 2020; 74 (2020):102258. 10.1016/j.janxdis.2020.102258. 32569905 PMC7286280

[32] Bakioğlu F, Korkmaz O, Ercan H. Fear of COVID-19 and positivity: mediating role of intolerance of uncertainty, depression, anxiety, and stress. Int J Ment Health Addict. 2020. Available from: 10.1007/s11469-020-00331-y. PMC7255700 32837421

[33] Moghanibashi A. Assessing the anxiety level of Iranian general population during COVID-19 outbreak. Asian J Psychiatr. 2020; 51: 102076. 10.1016/j.ajp.2020.102076 32334409 PMC7165107

[34] Lim GY, Tam WW, Lu Y, Ho CS, Zhang MW, Ho RC. Prevalence of depression in the community from 30 countries between 1994 and 2014. Sci Rep. 2018; 8(1): 2861. 10.1038/s41598-018-21243-x 29434331 PMC5809481

[35] Vlassoff C. Gender differences in determinants and consequences of health and illness. J Health Popul Nutr. 2007; 25(1): 47–61. 17615903 PMC3013263

[36] Lam J, Perales F. Chronic illness and mental strain: the moderating role of marital status over the disease cycle. Longit Life Course Stud. 2018; 9(3): 279–298. 10.14301/llcs.v9i3.483.

[37] Joensen LE, Madsen KP, Holm L, Nielsen KA, Rod MH, Petersen AA et al. Research: educational and psychological aspects diabetes and COVID-19: psychosocial consequences of the COVID-19 pandemic in people with diabetes in Denmark—what characterizes people with high levels of COVID-19-related worries? Diabet Med. 2020; 37(7): 1146–1154. 10.1111/dme.14319 32392380 PMC7273071

[38] Xiang Y, Yang Y, Li W, Zhang L, Zhang Q, Cheung T et al. Timely mental health care for the 2019 novel coronavirus outbreak is urgently needed. Lancet Psychiatry. 2020; 7(3): 228–229. 10.1016/S2215-0366(20)30046-8 32032543 PMC7128153

[39] Yao H. The more exposure to media information about COVID-19, the more distressed you will feel. Brain Behav Immun. 2020; 87: 167–169. 10.1016/j.bbi.2020.05.031 32413557 PMC7215146

[40] Smith G. Does gender influence online survey participation? A record-linkage analysis of university faculty online survey response behavior. ERIC Document Reproduction Service No. ED 501717: San José State University; 2008. Available from: https://scholarworks.sjsu.edu/cgi/viewcontent.cgi?article=1003&context=elementary_ed_pub.

[41] Folkman S, Greer S. Promoting psychological well-being in the face of serious illness: when theory, research and practice inform each other. Psychooncology. 2000; 9: 11–19. 10.1002/(sici)1099-1611(200001/02)9:1&lt;11::aid-pon424&gt;3.0.co;2-z 10668055

