# Supplementary material for: Levels and predictors of fear and health anxiety during the current outbreak of COVID-19 in immunocompromised and chronic disease patients in Saudi Arabia: A cross-sectional correlational study
Source: PLoS One. 2021 Apr 26;16(4):e0250554. doi: 10.1371/journal.pone.0250554 (PMC8075243; doi:10.1371/journal.pone.0250554)
Supplement: S1 Appendix — (DOCX) [file pone.0250554.s001.docx]

**Fear of COVID-19 Scale (English Version)**

Please respond to each item by ticking (√) one of the five (5) responses that reflects how you feel, think or act toward COVID-19.

| **Fear of COVID-19 Scale Item** | **Strongly disagree** | **Disagree** | **Neutral** | **Agree** | **Strongly agree** |
| --- | --- | --- | --- | --- | --- |
| I am most afraid of Corona |  |  |  |  |  |
| It makes me uncomfortable to think about Corona |  |  |  |  |  |
| My hands become clammy when I think about Corona |  |  |  |  |  |
| I am afraid of losing my life because of Corona |  |  |  |  |  |
| When I watch news and stories about Corona on social media, I become nervous or anxious |  |  |  |  |  |
| I cannot sleep because I’m worrying about getting Corona |  |  |  |  |  |
| My heart races or palpitates when I think about getting Corona |  |  |  |  |  |

Ahorsu DK, Lin CY, Imani V, Saffari M, Griffiths MD, Pakpour, AH. The fear of COVID-19 scale: development and initial validation. Int J Ment Health Addict. 2020. Available from: [https://doi.org/10.1007/s11469- 020-00270-8](https://doi.org/10.1007/s11469-%20020-00270-8).

**Fear of COVID-19 Scale (Arabic Version)**

يرجى الرد على كل عنصر بوضع علامة (√) على أحد الردود الخمسة (5) التي تعكس ما تشعر به أو تفكر فيه أو تتصرف به تجاه فيروس كورونا-١٩

| **أوافق بشدة** | **أوافق** | **لا أوافق و لا أعارض** | **لا أوافق** | **لا أوافق بشدة** | **عناصر مقياس الخوف من فيروس كورونا المستجد كورونا-١٩** |
| --- | --- | --- | --- | --- | --- |
|  |  |  |  |  | أنا خائف من فيروس كورونا-١٩لاقصى حد |
|  |  |  |  |  | التفكير بفيروس كورونا-١٩ يشعرني بعدم االرتياح |
|  |  |  |  |  | أشعر بتعرق في كفي عندما أفكر بفيروس كورونا |
|  |  |  |  |  | أخشى أن أفقد حياتي بسبب فيروس كورونا-١٩ |
|  |  |  |  |  | ينتابني القلق أو التوتر عندما أشاهد أخبا ًرا أو قص ًصا عن فيروس كورونا-١٩ |
|  |  |  |  |  | لا يمكنني النوم بسبب قلقي من اإلصابة بعدوى فيروس كورونا-١٩ |
|  |  |  |  |  | تسارع دقات قلبي عندما أفكر باإلصابة بعدوى فيروس كورونا-١٩ |

Alyami M, Henning M, Krägeloh CU, Alyami H. Psychometric evaluation of the Arabic version of the fear of COVID-19 scale. Int J Ment Health Addict. 2020. Available from: <https://doi.org/10.1007/s11469-020-00316-x>.

**The Hospital Anxiety and Depression Scale (English Version)**


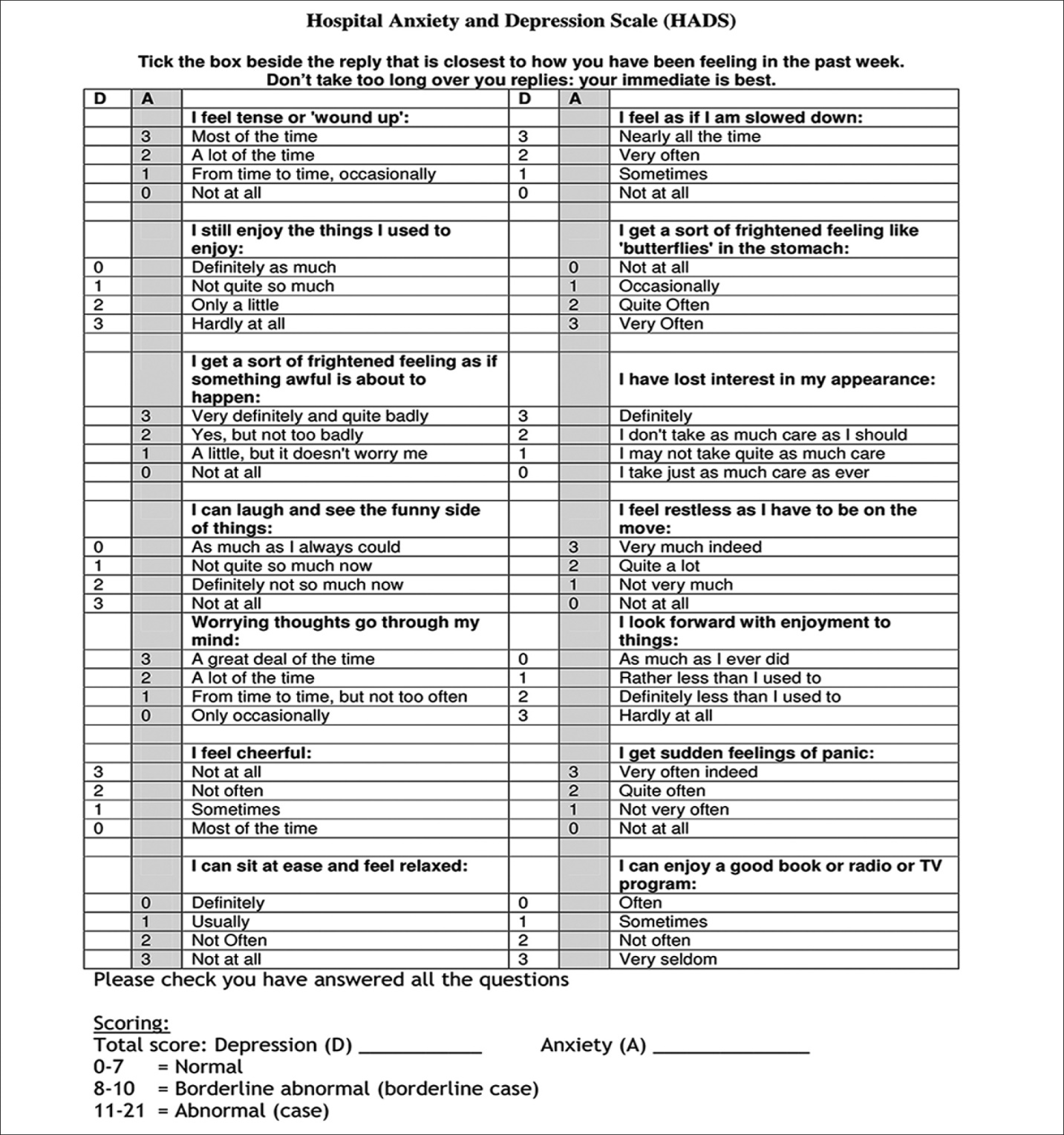


Zigmond AS, Snaith RP. The Hospital Anxiety and Depression Scale. Acta Psychiatr Scand. 1983; 67(6): 361–370. <https://doi.org/10.1111/j.1600-0447.1983.tb09716.x>.

**The Hospital Anxiety and Depression Scale (Arabic Version)**


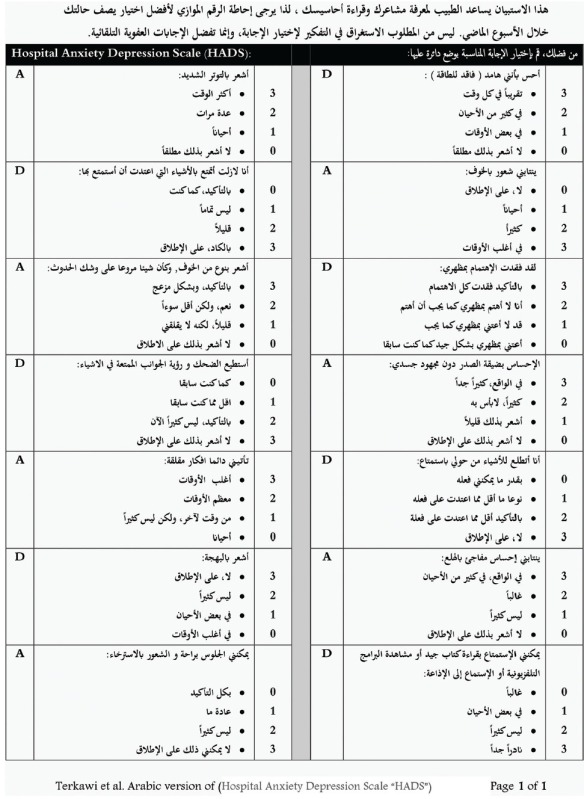


Terkawi AS, Tsang S, AlKahtani GJ, [AlKahtani](https://pubmed.ncbi.nlm.nih.gov/?term=AlKahtani+GJ&cauthor_id=28616000) GJ, [Al-Mousa](https://pubmed.ncbi.nlm.nih.gov/?term=Al-Mousa+SH&cauthor_id=28616000) SH, [Al Musaed](https://pubmed.ncbi.nlm.nih.gov/?term=Al+Musaed+S&cauthor_id=28616000) S et al. Development and validation of Arabic version of the Hospital Anxiety and Depression Scale. Saudi J Anaesth. 2017; 11(Suppl 1): S11-S18. <https://doi.org/10.4103/sja.SJA_43_17>.
